# Music Therapy for Delinquency Involved Juveniles Through Tripartite Collaboration: A Mixed Method Study

**DOI:** 10.3389/fpsyg.2020.589431

**Published:** 2020-10-23

**Authors:** Hyun Ju Chong, Juri Yun

**Affiliations:** ^1^Department of Music Therapy, Ewha Womans University, Seoul, South Korea; ^2^Ewha Music Wellness Research Center, Ewha Womans University, Seoul, South Korea

**Keywords:** juvenile delinquency, at-risk youth, music therapy, Young & Great Music Project, tri-partite collaboration, community networking

## Abstract

This study introduces a music therapy project for young offenders through community collaboration and its efficacy through a mixed method. The project called Young & Great Music is carried out via collaboration among three parties, which are the educational institution, the district prosecutor’s office, and corporate sponsor, forming a tripartite networking system. In this paper, we present an efficacy evaluation of the project’s implementation with 178 adolescents involved with the juvenile justice system: 115 youth was on suspension of indictment and 63 youth was under supervised probation. Quantitative and qualitative measures were collected and analyzed to examine the efficacy of the project. The music therapy program was developed for 15 sessions based on the use of music to prompt positive resources through music making and song writing. The efficacy was examined using three scales; self-concept, resilience, and stress coping skills. The paired *t*-test showed that there were significant improvement in all three scales respectively (*p* < 0.000). In order to examine the group difference between suspended indictment and supervised probation groups, Welch–Aspin *t*-test was conducted due to unequal variance of the group. Results showed there was a significant group difference in self-concept (*p* = 0.006) and resilience (*p* = 0.022). The study further examined participant’s experience of music and perceived benefits. Twenty participants had in-depth interviews about their music therapy experience which were recorded, transcribed and analyzed. Of the 109 statements derived from a qualitative content analysis of the interview transcripts, music making and song writing was repeatedly reported as helpful in gaining “new perspectives,” “courage to challenge and pursuit,” “perseverance,” and “self-acknowledgment.” The positive result of the study showed that the collaborative networking of regional and social resource to support for adolescents at-risk was successful. The results of this project are promising and suggest that other arts-based rehabilitation services and programs should be developed and implemented in juvenile justice system. For this, strategies for program sustainability for long-term facilitation are needed.

## Introduction

Adolescents who are involved in the juvenile justice system are commonly referred to as at-risk youth, defined as individuals who live with risk factors, such as personality traits, environmental characteristics, or family conditions, that have been shown to increase their likelihood of engaging in delinquency and other problem behaviors (Murray and Farrington, 2010). Developmentally at this stage, adolescents go through various emotional challenges from ecological, interpersonal or personal factors. If they have gained appropriate emotional skills to cope with negative moods and feelings, they learn to adapt to the social expectations. However, for adolescents living in the dysfunctional environment, often the emotions are either externalized through aggressive behavior or deeply internalized by repressing them. With such repression, emotional hyper or hyposensitivity, alexithymia, or any heavy feelings can elicit harmful or aggressive behaviors toward oneself and others (Engel-Yeger et al., 2016; De Berardis et al., 2017). Soon or later, if an appropriate rehabilitation therapy is not provided in time, such patterns of emotional coping can trigger any delinquent behaviors.

Among at-risk youth, many are the likely to be involved in criminal behaviors and depending on the type and frequency of the delinquent behavior, the juvenile justice system responds differently. Juvenile crime in South Korea is becoming a social issue, and rates of juvenile crime have risen in recent years. Although the number of juvenile offenders have reduced to 75,150 in 2018 from 84,026 in 2017 (10%), the offenders are becoming younger, more violent, and more likely to recommit crimes. In fact, one out of two offenders are convicted of different type of crimes, and the latency period is becoming shorter (Korean National Police Agency, 2020). The statistics show that over 25% of juvenile offenses are “accidental” crimes and nearly 10% are the result of curiosity (Supreme Prosecutor’s Office Republic of Korea, 2019). Without following up with rehabilitation, these youths can easily become reoffenders who commit more severe crimes, and moreover early involvement in the juvenile justice system would result in negative long-term outcomes for adolescents (Annie E. Casey Foundation, 2008).

In South Korea, when adolescents are charged with committing a crime for the first time, they are commonly given a 6-month conditional suspension of indictment, unless the nature of the crime is so severe as to warrant a harsher punishment. During this 6-month period, youth are required to complete a minimum number of hours of labor and community service. If they commit any type of crime during this period, they are likely to be prosecuted. Those who commit more severe crimes receive higher disposition, including supervised probation at a regional facility, where their daily schedule and whereabouts are continuously monitored and controlled. In other words, suspension of indictment is the lowest level response by the juvenile justice system and is often given to first-time offenders, while supervised probation is a higher response associated with more severe crimes and repeated offenses (Park, 2014).

Research shows that the arts have a positive impact on youth’s emotional and behavioral health (Cheliotis and Jordanoska, 2016). Especially for adolescents with emotional problems, the arts have been shown to serve as a therapeutic medium for communication (Ezell and Levy, 2003) and a safe space through which to release negative or excessive tension and energy (Kim et al., 2015). Arts can offer opportunities for creative self-expression in a contained and unthreatening environment, as any form or means of expression can be validated and respected. In one of the annual reports issued by the by the Western District Prosecutor’s Office in Seoul, Korea, they traced the recidivism rate among the youths with suspended indictment. The district attorney provided arts program instead of social labor hours to the youths with conditional suspension. After 1 year, it was reported that the recidivism rate decreased from 54% to 14% (Ewha Music Wellness Research Center, 2015).

Music as an art form has long been used as a therapeutic medium for youth involved in the juvenile justice system. Music has been used as a powerful catalyst promoting positive personal transformation through goal-oriented experience. For example, Genuine Voice was a short-term music learning program offered in a secure treatment facility (Baker and Homan, 2007). The program involved teaching musical composition and computer-based music sequencing to incarcerated youth. What was unique about this project was that it demonstrated the effect of lyric writing on personal expression. Through the writing process, the incarcerated youth learned to express various emotions, including forgiveness, healing, overcoming, and regret.

Good Vibration was another successful project for young offenders that provided opportunities to engage in creative music activities, such as composition, improvisation, and performance (Henley, 2015). It was a 1-week group project that leveraged intensive music experience to develop music-making skills. Music served as an outlet for emotional expression and self-actualization. Additional music programs targeting incarcerated youth include Music is the Answer in Israel, Music in Prisons in the UK, and Hip Hop Task Force in the United States (Baker and Homan, 2007).

Music can be used to address various emotional and psychological issues for youth involved in delinquency (Gold et al., 2017; Fairchild and McFerran, 2019; Santos, 2019). Various types of music therapy interventions have been offered to those in the juvenile justice setting, especially adolescents whose involvement in a criminal activity was due to a temporal misjudgment or impulsive behavior. It has been suggested that adolescents’ aggressive and impulsive behavior may derive from unresolved anger and impulse issues in the early stages of development (Turner et al., 2006; Grunwald et al., 2010). These issues may be the result of early experiences with victimization, dysfunctional family dynamics, unhealthy parenting, and trauma. Music is used to work with repressed unresolved emotions as a therapeutic outlet.

Among many therapeutic approaches for adolescents, positive psychology provides an appropriate rationale which emphasizes one’s resource rather than the problem itself. Based on the concept of positive psychology, resource-oriented therapy approach is formulated maximizing vital, creative and intrinsic motivation toward self-actualization using inner strength. Resource-oriented therapeutic interventions facilitate personal growth, expressive skills and ability to relate to others (Priebe et al., 2014). It is based on the premise that individuals have the potential to effectively deal with their own difficulties rather than passively being treated by somebody else.

Resource-oriented music therapy emphasizes the development and stimulation of a participant’s strengths and resources rather than the reduction of symptoms or cure of pathology. The focus is on the participant’s self-healing forces (Schwabe, 2005; Rolvsjord, 2016) via positive experiences, mastery, and coping rather than on difficult emotions, psychological conflicts, and problems.

The core features of resource-oriented music therapy involve four concepts (Rolvsjord, 2010). Firstly, it aims to nurture an individual’s strengths, resources, and potential. Resources are the center of attention and include musical competence. The development of such resources and strengths is connected to empowerment and experiences of positive emotions. The second concept is collaboration rather than intervention, taking the therapeutic relationship and self-authority of the participant into account. Third, the participant is viewed as an individual within their unique context, considering their social, structural, and cultural characteristics. This concept stresses the importance of interpersonal relationships. Lastly, music itself is seen as a resource. Therefore, it is important to provide diverse music experiences and opportunities so that the participants can access and maximize their strengths, resources, and potential in their internal world. Developing positive resource not only improves one’s self-concept but also reinforces one’s resilience of overcoming external life barriers and stress management (Schröder, 1997).

Collaboration is becoming a practical and essential concept in the fields of music therapy setting (Bolger et al., 2018). Collaboration is not only needed among the music therapy professionals but also at the community level. The core spirit of community of music therapy is networking and collaborating which means directly or indirectly working cooperatively with other professionals (Bolger, 2013; Stige and Aarø, 2012). Different countries have developed and provided arts-based rehabilitation or therapeutic programs for adolescents in prison, detention, and probation settings. Although a number of these programs are well-known music projects for at-risk youth, little has been reported on their long-term impact and effectiveness in reducing recidivism rates. Therefore, for sustainability of the project implementation, regional partners need to come together to form a collaborative network for the idea of rearing youth for the community.

The purpose of this study was to examine the efficacy of music therapy program provided through tri-partite collaboration on adolescents involved with the juvenile justice system and how the music therapy program helped the youths with their self-concept, resilience, and stress coping strategies. We also looked at whether differences existed between the participants with suspension of indictments and those under supervised probation. Lastly, we qualitatively examined the interview responses from a subgroup of participants to better understand the impact of the music therapy program on youth associated with the juvenile justice system.

## Materials and Methods

### Formation of Tripartite Networking Model of Young & Great Music Project

The Young & Great Music Project was initially developed in 2014 to bring three key stakeholders together for the common purpose of nurturing at-risk adolescents in the community. The three parties, which are the university, the district prosecutor’s office, and local corporations, came together to form a tripartite collaboration for implementation of music therapy services. These parties are invested in supporting community interests and caring for community members, including youth. It is important that current program utilized regional networking of core figures to ensure program sustainability.

Each party had distinctive role in the collaboration for the project facilitation (Table 1). Firstly, the university was responsible for providing the needed human resources (i.e., therapy expertise) for professional program development and The university’s faculty was willing to use the professional knowledge and practice for the society. Secondly, the corporate sponsor was responsible for providing the necessary financial support for the project. Lastly, the district attorney offered youth who were granted the 6-month suspension of indictment the option of completing community service hours or participating in the music therapy program. If they chose the music therapy program, they were required to attend and complete 15 music therapy sessions to fulfill the conditions of their suspension. Mentoring team under district attorney provided escorts to the participating adolescents to confirm the attendance as needed.

**TABLE 1:** Tripartite collaboration of Young & Great Music Project: partner’s roles.

Partner	District prosecutor’s office	University	Corporate sponsor
Agent	Juvenile Youth Department (Prosecutor)	Arts Therapy Department (Faculty and Graduates)	Social Contribution Department (Public Relations)
Roles	Recommend adolescents with conditional suspension of indictment for the program Connect with regional mentors if needed	Program development and implementation Provide supervisions Case management and report progress	Provide financial support for session fees, instruments, and technical support for music recording Provide spatial or physical resource for concerts

### Development of Resource-Oriented Music Therapy Program

The music project is formulated with music therapy interventions which utilizes music activities to achieve non-musical skills. In other words, music activities are formulated for personal and interpersonal goals using therapeutic music interventions. This is what differentiates music therapy from music learning or education. Music therapy sets the primary goal in achieving psychological and emotional well-being using music’s healing and therapeutic essence. Therefore, learning music comes only as a part of the process.

Young & Great Music Project is a 15-session program that is delivered in four stages: developing relationship with music and others and increasing self-awareness through the music experience (Sessions 1 through 3), identifying one’s resources and strengths (Sessions 4 through 8), identifying challenges and learning to use strategic resources (Sessions 9 through 12), and empowering one’s own inner strength through self-actualization experiences in music (Session 13 through 15). The session objective, content and music activities per stage are shown in Table 2.

**TABLE 2:** Resource-oriented music therapy for Young & Great Music Project.

Stage (sessions)	Session objective	Session content	Music activities
1st stage (1–3)	• Develop relationship with music and others in the group • Promote self-awareness in musical environment	• Explore various music and establish connection • Challenge musical experiences: listening, singing, and playing • Share one’s feelings and thoughts about music	• Music/song listening • Lyric discussion • Instrument learning: guitar, drum, or keyboard
2nd stage (4–8)	• Explore inner resources • Develop music repertoire for positive resource	• Learn to express positive moments and personal likings about the music • Explore new ideas that stand out in musical themes • Write or rewrite lyrics to express and dialogue with oneself	• Thematic music listening • Song discussion and lyric rewriting • Thematic music lists • Vocalization/singing • Instrument playing: guitar, drum, or keyboard
3rd stage (9–12)	• Identify personal challenges • Develop strategic resource using music	• Explore any difficulties in the music-making process • Come up with personally meaningful song: “my grounding song” • Discuss how the song/materials may help	• Listing supportive music/“power music” • Song lyrics for grounding • Instrument playing: guitar, drum, or keyboard
4th stage (13–15)	• Experience self-actualization in music • Empower inner strength through music	• Record the original songs with own voices • Perform on stage and allow their voices to be heard by others	• Instrument playing of repertoire • Singing and recording: solo, duet, and chorus • Rehearsal for the concert

### Participants

The participants were recruited from the project implemented in 2017–2018. From 215 adolescents, 178 who have successfully attended all 15 sessions and completed both pre and posttests participated. The participants were from two types of groups. One group had received conditional suspension of indictments (SI) and the other group was under supervised probation (SP) in an independent facility in the district. From SI group, 115 adolescents participated, and from SP group, 63 adolescents participated. Among 178, 163 were male and 15 were female. The average age was 17.5 years (Table 3). The total number of music therapy groups was 42, each group having 4–5 participants. The groups were formed considering their schools, relationship issues among them, and the timing of referral from the district prosecutor’s office.

**TABLE 3:** Number of participants and music therapy session groups.

Factor	Suspended indictment (SI)		Supervised probation (SP)		Total
	Male	Female	Male	Female	
Participants (*N*)	103	12	60	3	178
Age (*SD*)	17.3 (1.05)	17.3 (0.92)	17.9 (1.24)	17.3 (0.92)	17.5 (1.14)
MT session groups	28		14		42

For the SP group, their participation was voluntary. Because the music therapy sessions were held in the facility in which they resided, they could make the decision whether to take part or stay in their room. Once they agreed to participate, however, they were encouraged to attend all 15 music therapy sessions for group consistency.

Before the implementation of the program, orientation day was held to introduce about the music therapy program and its content. Adolescents, guardians, facility staff, mentors under the district prosecutor’s office were all invited to the orientation. Participant consent was received on the program orientation. The details of the program were communicated to the participants and their legal guardians before signing the informed consent on the participation and video/audio recordings of the session. Due to the nature of the study and the vulnerability of the participants, the entire program implementation and data collection procedures were strictly examined and reviewed by the district attorneys and university research team.

### Project Implementation

The implementation procedure was as follows: The district attorney in charge of juvenile youth contacted the music therapy team at the university when an adolescent with suspended indictment opted to take part in the music therapy program instead of community service hours. Once the number of referred adolescents reached four or five, a music therapy group was formed.

For adolescents at the supervised probation facility, music therapy sessions were provided in the equipped music room, which housed the music instruments funded by the corporate sponsor. For the suspended indictment group, local mentors from the district attorney escorted the participants from the suspended indictment group and recorded their attendance which was required as part of the youths’ conditional conviction. The music therapy sessions were offered at the university facility fully equipped with instruments and recording devices.

As the final step, following the 15-week session program, the university facilitated a small concert at the regional art or concert hall where the participants sang their original songs and showed the music competency they had built during the program. This concert was funded by the corporate sponsor and was open to the public when the participants agreed (Table 4).

**TABLE 4:** Implementation process.

Participant groups	Motives	Referral	Attendance	Music therapy program
Suspension of Indictment (SI) Group	Opted to take part in music therapy program instead of community labor or social service work	Juvenile Youth Team, District Attorney	Attendance needed to release from conditional suspension	MT team provides music therapy orientation about the objective and content of the music program before its beginning.
Supervised Probation (SP) Group	Volunteer to take part for music experience	Supervisor of Probation Facility upon the participant’s wish	Attendance is not mandatory but encouraged to participate in the program	Participants attend 15- weekly sessions. MT team administers quantitative measurements before and after the program. MT team holds interview with volunteered participants about the music experience.

### Data Collection and Analysis

Data were collected using two different types of measures: quantitative and qualitative measures. In terms of the quantitative data, three scales were used to assess the efficacy of the music therapy program: participant’s self-concept, resilience, and stress coping strategies before and after the program. In the beginning of the program, the scales were distributed for pretest. After administering 15-week program, posttests were distributed to those who successfully completed 15-week program.

For qualitative data, following the completion of the program, additional interviews took place in order to examine participant experience regarding the perceived benefits of music. The interviews were carried out individually by the researchers for about an hour. Their interviews were recorded with consent. The procedures of participant recruitment, data collection, and interview process were reviewed and approved by the Institutional Review Board at the Ewha Womans University (IRB No. 125-14).

#### Quantitative Data Analysis

Self-concept: Self-concept was measured by the Self-concept Scale developed by Lee (1997), which consisted of 30 items that evaluated an individual’s physical and personal features from one’s own and other’s perspectives based on five-point Likert-type scales. The Cronbach’s alpha was 0.90, and higher scores meant more positive self-concept.

Resilience Scale of Korean Adolescents: Resilience was measured by the Resilience Scale of Korean Adolescents by Lee and Jo (2005). The self-report type questionnaire consisted of 48 items that evaluated intrapersonal characteristics related to cognitive, emotional, intentional, and spiritual resilience and external protective factors associated with family, school, community, and peer group psychosocial resources. The tool consisted of five-point Likert-type scales, and the Cronbach’s alpha was 0.91. Higher scores indicated higher levels of resilience.

Stress coping strategies: The Korean version of the Stress Coping Strategies (Kim, 1987), originally developed by Lazarus and Folkman (1984), was used to measure each participant’s level of stress coping strategies. The questionnaire included 27 five-point Likert type items associated with sub-factors of problem-focused coping, emotion-focused coping, meaning-making coping and support-seeking coping. The Cronbach’s alpha was 0.84, and higher scores indicated more positive stress coping strategies.

To compare changes in measures of three scales from before the program with those taken after the program, paired *t*-test was used. To examine the group difference between SI and SP groups, Welch–Aspin *t*-test was conducted due to the unequal variance between the groups.

#### Qualitative Data Analysis: Interviews

Twenty participants volunteered to participate in interviews after the completion of their 15-week music therapy program. Their individual interviews were arranged separately after the completion of other tools. The participants were asked to openly express any thoughts and feelings about the program and freely describe how music helped them if it was deemed helpful. The semi-structured questions allowed the participants to freely articulate whatever came to mind about their experience. The researchers who did not provide music therapy sessions carried out the interviews in order to hold neutral stance in the evaluation of the program implemented.

Qualitative data were analyzed using the qualitative content analysis method. Content analysis is a method of inductive analysis in which researchers continuously compare data to systematically derive meaningful units and categories. Qualitative content analysis is a method for organizing descriptive responses into the meaning segment of qualitative data. This is done by assigning successive parts of the material to the categories of a coding frame (Mayring, 2000; Schreier, 2012, 2014). The collected data were transcribed and statements were drawn for further categorization. The categorized themes were counted in order to verify the weight of experiential essence verbalized by the participants.

## Results

### Analysis of Quantitative Data

The study conducted paired *t*-test to examine the pre to post changes after the program. Results showed that there were significant differences in all three measures: self-concept (*p* < 0.000), resilience (*p* < 0.000) and stress coping strategies (*p* < 0.000) (Table 5). Further the study examined if there were differences in the changes between the groups, SI and SP. As the group showed unequal variance, Welch–Aspin *t*-test was conducted. Among the three scales, result showed significant differences between the SI and SP group in self-concept (*p* = 0.006) and resilience (*p* = 0.022), and no difference in stress coping strategies (*p* = 0.061). The study further examined the effect size. According to the Cohen’s guidelines (Cohen’s, 1988), the difference between the group was large in self-concept (*d* = −0.59) and moderate in resilience (*d* = −0.49). In other words, the participants in SP group showed significantly greater changes in two scales than SI group (Table 6).

**TABLE 5:** Comparison of pre- and post-scores of self-concept, resilience, and stress coping strategies (*N* = 178).

Dependent variables	Pre-test *M (SD)*	Post-test *M (SD)*	*t*(*p*)
Self-concept	3.36 (0.432)	3.54 (0.493)	−5.928 (0.000)
Resilience	3.38 (0.432)	3.57 (0.515)	−5.726 (0.000)
Stress coping strategies	3.30 (0.408)	3.50 (0.463)	−6.131 (0.000)

**TABLE 6:** Comparison analysis between suspended indictment (SI) group and supervised probation (SP) group.

Variable	Group	Pre-test *M* (*SD*)	Post-test *M* (*SD*)	Post–Pre *M* (*SD*)	*t*(*p*)
Self-concept	SI (*n* = 115)	3.38 (0.421)	3.49 (0.468)	0.112 (0.327)	−2.799 (0.006)**
	SP (*n* = 63)	3.33 (0.452)	3.64 (0.525)	0.312 (0.512)	
Resilience	SI (*n* = 115)	3.41 (0.431)	3.54 (0.511)	0.129 (0.365)	−2.327 (0.022)*
	SP (*n* = 63)	3.33 (0.432)	3.65 (0.518)	0.313 (0.566)	
Stress coping strategies	SI (*n* = 115)	3.30 (0.404)	3.45 (0.438)	0.152 (0.372)	−1.898 (0.061)
	SP (*n* = 63)	3.29 (0.419)	3.59 (0.496)	0.298 (0.543)	

### Analysis of Qualitative Data

The interview responses of 20 participants regarding their experiences in the music therapy program were recorded and transcribed. Using the content analysis method, researchers examined what were the types of resources and benefits that participants experienced in the music therapy program. After repeatedly reviewing the interview transcripts to delineate common themes regarding the participants’ music experience, five themes were identified. The themes are operationally defined as categories based on the operational definitions accordingly (Table 7).

**TABLE 7:** Types and operational definitions of inner resources experienced by the interviewed participants.

Nature of inner resource	Operational definitions
New perspectives on life	Developing new and positive perspectives regarding oneself, relationships with others, and current or future circumstances
Courage to challenge and pursuit	Intrinsic motivation and strength to overcome life’s hardships and adversity Seeking and developing strategies and making effort to achieve goals
Perseverance	Enduring difficult moments and able to tolerate moments of tension and conflict. Striving to pursue continuously
Meanings for accomplishment	Identifying value and meaning in the work completed and mastery Being able to be content with one’s own effort and achievements
Self-acknowledgment	Validating oneself as a unique person Accepting oneself and learning to be more genuine

Based on the operational definitions of the initial categories derived from the data, participants’ statements were sorted accordingly. All participants provided multiple statements regarding the benefit of their music experience. Total statements drawn were 109, and these were categorized by the nature of the inner resource (Table 8). Among the five categories of resources, 49 statements were about new perspectives on life. These participants described becoming a different person and living a different life, breaking out of the negative thought patterns they used to have. Their wishes and wants were related not only to their own life but to significant others, such as parents, family members, and friends.

**TABLE 8:** Type of inner resources reported by the interviewed participants.

Resource	Open coding samples	N (%)
New perspectives on life	• Wanted to stop feeling regretful and write a song about the new person they were becoming • Wanted to become someone who was skillful in something • Wanted to express appreciation and apologize to parents • Wanted their song to console someone who was in a similar situation as them • Wished the song would shed light on others who were struggling • Wanted all the depressive and anxious feelings to fly away • Wanted to live an ordinary life like other people • Wanted to overcome thinking they were insignificant	49 (45%)
Meanings for accomplishment	• Think the biggest benefit is that I gained the ability to write songs and play instruments • Love the fact that I completed and created something on my own • Thought, “Now, I do have a skill” as I was writing songs and playing music • The best part was that I learned something new • Think (the program) helped us to attain skills • The best part is that we really completed songs, although we doubted the possibility at first • Gained performing skills that can be useful elsewhere	21 (19.3%)
Courage to challenge and pursuit	• Because I was brave here, I think I can be more courageous when doing things in the future • The experience here will help us to become more courageous; people like us do need a lot of courage when trying new things • Because I was able to sing in front of others here, I think it will help me become more courageous and try new things • While making the songs, I needed the courage to share something about me with other people	18 (16.5%)
Perseverance	• Learned that withstanding and enduring difficult times eventually make things work • Learning to play musical instrument was difficult at first, but when I persisted, it got better and I was able to complete it • Feel like I have gained patience that I never had • Kept thinking that I cannot give up • Doing something with strangers was difficult at first, but things became comfortable as I did not give in • It was a bit boring and annoying as I couldn’t do it well in the beginning, but as I kept trying, I learned how to endure. • It was my first time trying out something steadily	14 (12.8%)
Self-acknowledgment	• Became more aware of how my mind is through the song • Thought the song is me because I tried to express my mind through the song • Song was like a window through which I could look into my heart • Singing my own song touched my heart, which felt like I was caressed • Song is the story of my inner mind, story about something that I really want to do or want to have.	7 (6.4%)
Total		109 (100%)

Statements regarding the meaning of accomplishments rated second (21 statements). These participants expressed joy about making their song lyrics and melodies. They were content not only with the quality of the music they mastered, but with the fact that they did not give up.

Participants also expressed gaining courage to challenge themselves and pursue their life goals (18 statements). Because the music experience provided in the program was new for most of the participants, singing and recording their own song to share with others required courage and effort. There were also statements that revealed perseverance and self-acknolwedgement. They willingly endured to master their musical work (14 statements). At the same time, some participants used the music making moments comming in contact with new thoughts, feelings, emotions and wishes (7 statements).

## Discussion

The purpose of this study was to examine the efficacy of a music therapy program called Young & Great Music Project for youth involved with the juvenile justice system provided through tri-partite collaboration in the community. The study showed that the participants in the music therapy program have gained positive self-concept, resilience and stress coping strategies during the 15-weekly sessions. The outcome of the study support the existing literature that music offered the opportunities to come in contact with their inner potentials and strength (Gold et al., 2017; Fairchild and McFerran, 2019; Santos, 2019). The outcome of this study further reinforce the use of arts as rehabilitative therapy medium for healing and reducing youth recidivism (Cheliotis and Jordanoska, 2016).

There are a few important implications from this study. Firstly, music provide vicarious experience for self-value through music making and playing. Musical tasks in the program reinforced a sense of control, the ability to make good choices, ownership of the music repertoire, and self-actualization through music making. This kind of self-ownership and self-authorship are two important components that can replace unresolved needs and fulfillment of a distorted identity, which otherwise were dealt with by aggressive means (Olson-McBride and Page, 2012). It was shown that adolescents often commit crimes to confirm their existential sense of self (Lee, 2013). In this aspect, the resource-oriented concept in the program was well in-tune with the participants.

In the same line, the interviewed statement showed that song writing prompted the participants to seek new perspectives and challenges. Among the various music interventions, lyric writing was described as the most successful way for the participants to access their unexpressed thoughts and desires, which led to making life decisions (Krüger and Stige, 2015). These experiences served as opportunities for greater self-exploration and to gain deeper self-awareness.

Secondly, both the quantitative and qualitative data showed that this program serves to be a pivoting moment for the participants, especially those with conditional suspension of indictment (SI group). The program was deemed as a ‘condition’ to waive the indictment, however, ironically this serves as an opportunity to heal themselves and go through major changes in their attitudes and thinking. In other words music therapy was given as a conditional obligation to fulfill the conditional suspension, however, most participants became to enjoy the music experience. Upon the termination of the program, some of them inquired about joining the program again.

Third, despite the fact that both groups showed significant improvement in self-concept, resilience and stress coping strategies, the supervised probation group showed greater changes. This may imply that the self-choice may have offered sincere and genuine personal work. Unlike the SI group, they volunteered to take part without any conditional obligation. Residing in the supervised facility may be rather limiting to explore something new and creative. Music may have offered them opportunities to meet these needs. Such effort is an indication of music prompting intrinsic motivation for better self, bringing a renewed attitude toward new experiences and openness (Daykin et al., 2013; Travis, 2013). These new sentiments and perspectives were evident in the interviews.

Fourth, the results of these study suggest important implications on how practical and utilizing aspect of music’s benefit should be facilitated via community collaboration. The spirit of this music project was that it was provided through the collaborative work of regional agents among university faculty, corporate sponsors, attorneys and staff from the district prosecutor’s office. The corporate involvement as a sponsor is crucial for sustainability of the project. The university’s commitment to share its knowledge with the larger community is similarly essential. Most importantly, district attorney subscribing to the mindset of providing help and healing to youth rather than punishment is also necessary, as they are the first party to encounter youth caught up in the juvenile justice system. The therapy opportunity the adolescents received despite of their offending conduct, brought a turnaround moment in their life. The shared goals and perspective of the tripartite enabled the project to continue on for the past 7 years in South Korea. Without this common purpose, the project would have been one-and-done effort with no lasting impact.

There are a few caveats of this research. As this project and research was carried out in a real world setting through community networking and collaboration involving those in the juvenile justice system, it was not controlled for optimal clinical trial condition. This means that one should keep in mind that the efficacy outcome derived from this study is based on within group pre-posttest design. For more in-depth findings of music’s therapeutic effect, random control trial design may be sought in the future studies.

Secondly, another caveat of this study is that therapist-related variable may have affected the result. Since there were more than one music therapist leading the session groups, therapists’ individual traits may have affected the outcome of the overall project efficacy. For youth involved in delinquency, interpersonal interactions are sensitive issue therefore, therapist quality and style can be an important factor that affect their motivation and level of participation. In this project, regular supervisions were provided to the therapists and the project outcomes are positive, however, therapist-related factor should be always considered in further studies as well.

Lastly, it would be essential to examine how music’s benefit continued in their lives after the completion of the program. A follow-up study to examine the maintenance effect of the music therapy program after certain period would strengthen music’ effect and further support the tri-partite networking and collaboration. Through examining the long-term impact of the collaboration, the networking can be systemized among the three parties as a model.

As a conclusion, there is an old proverb that states, “It takes a village to raise a child.” This applies to societies as well. When it comes to at-risk youth exhibiting delinquent behavior, it is for the community partners to come together and collaborate (Chong et al., 2020). It is insufficient to conceptualize at-risk youth as solely a familial issue. Instead, regional agents can play supportive and helping roles in providing guidance and support so the adolescents can become contributing members of society.

## Data Availability Statement

The datasets used to generate the results for this study can be obtained from the authors when deemed necessary.

## Ethics Statement

The studies involving human participants were reviewed and approved by Ewha Womans University Institutional Review Board. Written informed consent to participate in this study was provided by the participants’ legal guardian/next of kin.

## Author Contributions

HC, as the PI of the proejct, designed the study. HC and JY developed the therapeutic program. JY implemented the program and collected data. Both authors contributed in the process of drafting the manuscript.

## Conflict of Interest

The authors declare that the research was conducted in the absence of any commercial or financial relationships that could be construed as a potential conflict of interest.
