# Corrigendum: Music Therapy for Delinquency Involved Juveniles Through Tripartite Collaboration: A Mixed Method Study

**DOI:** 10.3389/fpsyg.2020.641540

**Published:** 2021-01-12

**Authors:** Hyun Ju Chong, Juri Yun

**Affiliations:** ^1^Department of Music Therapy, Ewha Womans University, Seoul, South Korea; ^2^Ewha Music Wellness Research Center, Ewha Womans University, Seoul, South Korea

**Keywords:** juvenile delinquency, at-risk youth, music therapy, Young & Great Music Project, tri-partite collaboration, community networking

An author name was incorrectly spelled as Hyun J. Chong. The correct spelling is Hyun Ju Chong.

The authors apologize for this error and state that this does not change the scientific conclusions of the article in any way. The original article has been updated.

